# Molecular evolution and expression patterns of myxovirus resistance proteins in *Lampetra japonica*: Evolution and expression of lamprey myxovirus resistance protein

**DOI:** 10.3724/abbs.2024019

**Published:** 2024-02-23

**Authors:** Jinzhao Liu, Meiyao Chu, Jiahui Kuang, Xinran Wang, Yijie Zhang, Lutian Wang, Yimeng Xia, Yifan Sun, Xinxin Liu, Jing Li, Jun Li, Ting Zhu

**Affiliations:** 1 College of Life Science Liaoning Normal University Dalian 116081 China; 2 Lamprey Research Center Liaoning Normal University Dalian 116081 China; 3 Collaborative Innovation Center of Seafood Deep Processing Dalian Polytechnic University Dalian 116081 China

The myxovirus resistance (Mx) protein has strong GTP phosphohydrolase (GTPase) activity and is a member of the evolutionarily conserved GTPase dynamin protein family. The Mx protein sequence is divided into three parts: the amino-terminal GTPase domain, the central interactive domain (CID), and the carboxyl-terminal GTPase effector domain (GED) with a leucine zipper motif [1]. The GTPase domain, a relatively conserved part of the Mx protein and other members of the dynamin protein family, is composed of approximately 300 amino acids. It contains three conserved GTP-binding motifs (GDXXSGKS, DLPG, and TKPD) and a tag (LPRXXGXXTR). The first two GTP-binding motifs are linked to the side of the tag and bind to the phosphate moiety of GTP, whereas the third GTP-binding motif is highly important for its binding to guanosine [ 2, 3]. When cells are infected by a virus, exposed to interferon, or stimulated by double-stranded RNA, the activation of *Mx* gene transcription significantly increases *Mx* mRNA expression [ 4 ‒ 6], which is translated into the Mx protein [ 7 , 8] and performs antiviral functions.

*Lampetra japonica* is the most primitive marine vertebrate and an ideal model for studying embryonic development, organ differentiation, and immune system evolution in vertebrates. However, the adaptive immune system of lampreys is not fully developed. Lampreys use immune factor-variable lymphocyte receptors and rely primarily on innate immunity to resist pathogenic microorganisms [ 9, 10]. Although the antiviral activity of Mx proteins has been widely studied in mice, jawed fish, and other jawed vertebrates, the origin, evolution, and antiviral activity of these proteins in lamprey, a lower vertebrate without jaws, have not been properly elucidated. Therefore, in this study, we aimed to investigate *Mx* genes in *L*. *japonica*.

Full-length sequences of the Lj- *MxA* and Lj- *MxB* open reading frame (ORF) regions were successfully cloned from *L*. *japonica* (the primers used are listed in Supplementary Table S1). The ORF of Lj- *MxA* has 1959 base pairs which encodes a protein of 652 amino acids with a predicted molecular weight of ~73.27 kDa and a theoretical isoelectric point of 5.85. The ORF of Lj- *MxB* has 2193 base pairs which encodes a protein of 730 amino acids with a predicted molecular weight of ~81.76 kDa and a theoretical isoelectric point of 6.85. It was predicted that the secondary structures of the Lj-MxA and Lj-MxB proteins would meet the typical characteristics of the Mx protein sequence and that they also have three typical domains of dynamin GTPases [ 11, 12]: the GTPase (G) domain for binding and hydrolyzing GTP, three bundle signaling elements (BSEs), and the stalk region ( Supplementary Figure S1). The structural difference between Lj-MxA and Lj-MxB is a region of 40 amino acids in the disordered loop L4 protruded from the compact stalk ( Supplementary Figure S1). This loop is similar to the PH domain of the dynamin protein and has been shown to mediate membrane interactions and viral target recognition. Various vertebrate and invertebrate species were selected for multiple sequence alignment. Lj-MxA and Lj-MxB are 90.49% similar, and their similarity to the Mx protein sequences of other species ranges between 40.34% and 67.89%. The Lj-MxA and Lj-MxB sequences are highly conserved in the N-terminal GTPase triad GTP-binding region “GDQSSGKS”, “DLPG”, “TKPD”, and the tag “LPRGSGIVTR”, which is consistent with the structural characteristics of this protein family and indicates that the Lj-MxA and Lj-MxB sequences and structurally conserved domains participate in similar pathways. The C-terminus is a highly conserved leucine zinc finger region similar to that in other species and is related to the activation and functional coordination of GED and is necessary for antiviral activity. Lj-MxA is primarily localized in the cytoplasm, whereas Lj-MxB is present in both the cytoplasm and the nucleus.

To understand the evolutionary relationships of Mx from lamprey, a phylogenetic tree was plotted using the neighbor-joining method ( Figure 1A), and motif prediction was performed ( Figure 1A) using known Mx protein sequences of different species retrieved from the NCBI ( Supplementary Table S2). According to the phylogenetic tree with *Saccoglossus kowalevskii* Mx as the outgroup, Lj-MxA and Lj-MxB, together with similar proteins from hagfish and sea lamprey, are in the intermediate stage from invertebrates to vertebrates. We observed that the fish Mx proteins *Lepisosteus oculatus* Mx1 (Lo-Mx1), *Danio rerio* (Dr-MxA), *L* . *oculatus* Mx2 (Lo-Mx2) and *D*. *rerio* MxC (Dr-MxC), as well as *L*. *oculatus* Mx3 (Lo-Mx3) and *Callorhinchus milii* Mx1 (Cm-Mx1), were clustered together. It is speculated that before species differentiation, different fish ancestors experienced gene duplication and type differentiation within the species to adapt to environmental changes, but the paralogs retained the original characteristics of Mx, as is observed in birds, amphibians, reptiles, and mammals.

**Figure 1:**
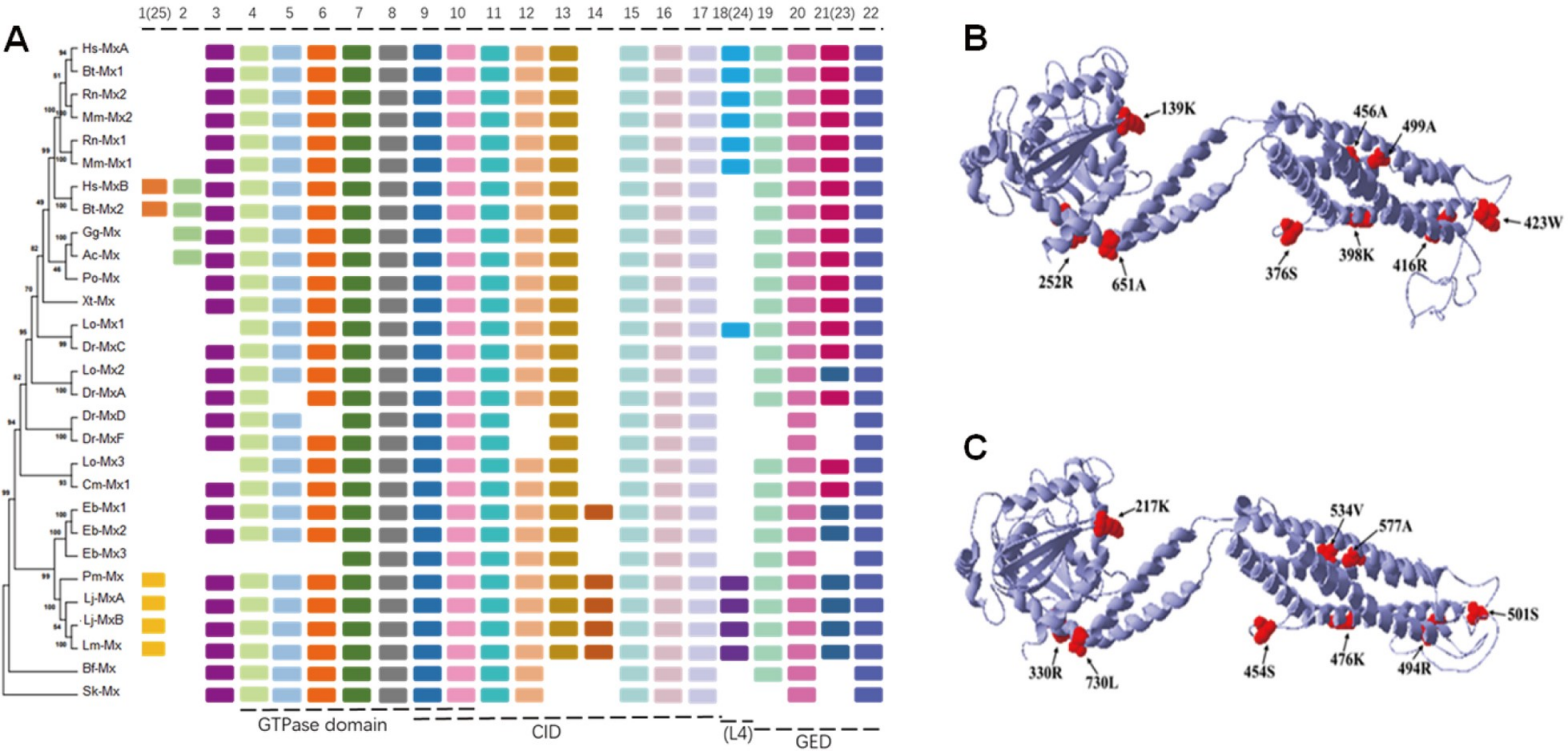
Figure 1 Phylogenetic tree and positive selection sites on *L. japonica* MxA and MxB (A) The evolutionary history of MxA and MxB was inferred from a phylogenetic tree constructed using the neighbor-joining method with 1000 bootstrap repetitions. The evolutionary distance was calculated using the JTT matrix-based method in MEGA7. The table on the right represents the type and distribution of conserved motifs discovered among the Mx sequences using the MEME tool. Each colored box represents a specific conserved domain. GED, GTPase effector domain; CID, central interactive domain. (B,C) Tertiary structure prediction of Lj-MxA and Lj-MxB. The arrow indicates the locations of all the detected positive selection sites on the tertiary structure of Lj-Mx.

The predicted number of Mx motifs in multiple species, including lamprey, is 25. The Mx motif of each species has relatively conserved evolutionary characteristics. Most Mx proteins have motifs 4–10 that collectively constitute the GTPase domain. Motifs 9–18 and 24 may jointly form the CID; motifs 19–23 jointly form the effector region GED of GTPase ( Figure 1A). However, there are some noteworthy differences in the motif compositions between various species. First, compared to those of the *Branchiostoma* *floridae* and *S*. *kowalevskii* Mx proteins, motifs 25, 13, 14, 24, and 23 appear in lamprey, suggesting that its motif composition is more similar to that of higher vertebrates. The *Mx* gene of lamprey may be the ancestral gene of vertebrates. Second, motifs 24, 23, and 25 in lamprey are analogous to motifs 18, 21, and 1, respectively, in higher vertebrates. Interestingly, proteins containing motif 18, such as *Homo sapiens* MxA, *Bos taurus* Mx1, and Mx1 and Mx2 in rodents, have been shown to exert antiviral effects. We speculate that the different motif compositions of the lamprey proteins can be explained by adaptation to complex aquatic environments. However, motif 14 is unique to lamprey and hagfish, and as a component of the CID, it may be related to the particular immune signaling response in cyclostome.

Evolutionary events occur frequently but selectively in nature. The evolutionary rates of the Mx proteins of different species (except lamprey) in the free model are less than 1 according to the branch model analysis (ω=dN/dS), suggesting that Mx may be generally constrained by selection pressure. During the evolutionary process, positive selection can act on a small number of amino acid sites, and a site model test was conducted to verify this phenomenon. Likelihood ratio tests between the two nested models (M3 vs M0 and M8 vs M7) revealed that an alternative hypothesis that allowed for positive selection fit the data significantly better than neutral models. According to the Bayes Empirical Bayes analysis results, three positive selection sites were identified by the M8 model (posterior probability greater than 0.95) ( Supplementary Table S3). In the branch model, ω>1 was used for sea lamprey. It has been speculated that the *Mx* gene of lamprey may be driven by positive selection. To determine the evolutionary driving force of the *Mx* gene in lamprey species, we further evaluated the evolutionary driving force of the foreground branch compared to that of the background branch and confirmed the positive selection sites. The results showed that there could be 6 significant positive selection sites in the MxA and MxB proteins of lamprey ( Supplementary Table S3).

Combining the results of the site model and branch-site model, in lamprey Lj-MxA and Lj-MxB, there may be 9 positive selection sites located in the following elements of the tertiary structure: sites 139K and 252R of Lj-MxA are in the GTPase domain; sites 376S, 398K, 416R, 423W, 456A, and 499A are in the CID of the stalk; and site 651A is located in a BSE ( Figure 1B). In Lj-MxB, sites 217K and 330R are in the GTPase domain; sites 454S, 476K, 494R, 501S, 577A, and 534V are in the CID of the stalk; and site 730L is in a BSE ( Figure 1C). Compared with those of other dynamin proteins, the amino acid residues of the GTPase domain of Mx proteins have a unique amino-terminal extension. We speculate that the two positive selection sites in the GTPase domains of Lj-MxA and Lj-MxB may be involved in adaptive evolution and may mediate several special functions. CID and post-folding of the LZ region in the GED increase GTP enzyme activity, which is necessary for recognizing virus-specific sites. In Lj-MxA and Lj-MxB, although most of the positive selection sites are located in the CID, no positive selection sites are found in the GED.

Using quantitative real-time polymerase chain reaction (qRT–PCR) analysis of total RNA samples, *MxA* and *MxB* expression levels were studied in seven *L*. *japonica* tissues: liver, gill, kidney, intestine, heart, leukocytes, muscle, and supraneural body. Lj- *MxA* and Lj- *MxB* were expressed in all these tissues. Lj- *MxA* and Lj- *MxB* were highly expressed in the heart and gill tissues of lamprey, whereas their expression levels in the intestine, kidney, supraneural body, liver, muscle, and leukocytes were lower ( Figure 2A). These results showed that Lj-MxA and Lj-MxB may participate in the immune defense of lamprey and play major roles in heart and gill tissues.

**Figure 2:**
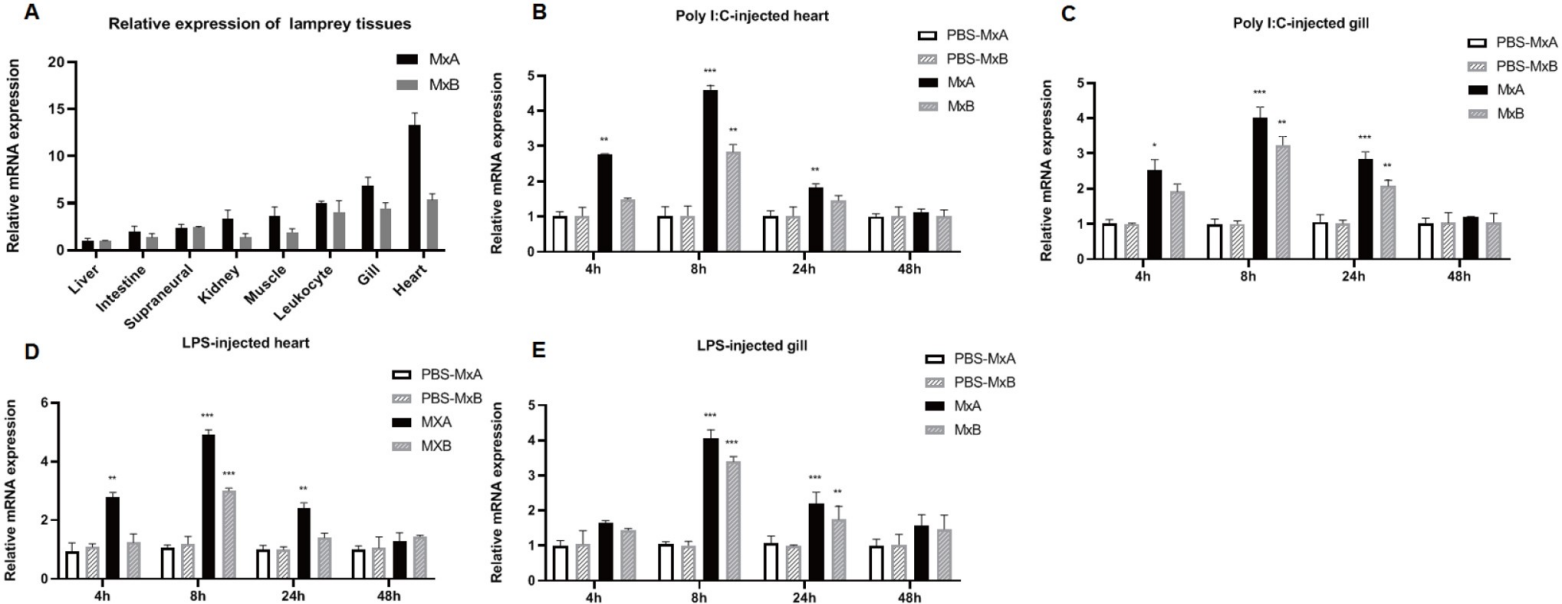
Figure 2 Expression patterns of Lj- *MxA* and Lj- *MxB* (A) Relative expression levels of Lj-MxA and Lj-MxB in different organs and tissues of lamprey. (B,C) Changes in the relative expression levels of Lj- MxA and Lj-MxB in the heart (B) and gills (C) of lamprey after challenge with Poly I:C. (D,E) Changes in the relative expression levels of Lj-MxA and Lj-MxB in the heart (D) and gills (E) of lamprey after challenge with LPS. Data are presented as the mean±standard error of the mean (n=3). The statistical significance of the differences in Lj-Mx mRNA expression between the stimulated and corresponding control groups are indicated with asterisks as follows: *P<0.05, **P<0.01, and ***P<0.001.

The immune functions of Lj-MxA and Lj-MxB stimulated by different pathogen-related molecular models were analyzed via qRT-PCR, with the *gapdh* mRNA expression level as the internal control. The expression levels of Lj- *MxA* and Lj- *MxB* were determined following treatment with polyinosinic:polycytidylic acid (Poly I:C) and lipopolysaccharide (LPS). After Poly I:C stimulation, in both the heart and gills, the expression of Lj- *MxA* was initially upregulated significantly at 4 h ( *P*<0.01 and *P*<0.05 in the heart and gills, respectively) and peaked at 8 h ( *P*<0.001); however, these levels decreased at 24 h and reached the levels observed in the PBS-injected controls at 48 h ( Figure 2B,C). After stimulation with LPS, both the Lj- *MxA* and Lj- *MxB* genes exhibited a high degree of similarity in their response to Poly I:C stimulation; however, the expression levels of both genes in the gills did not increase significantly at 4 h ( Figure 2D,E). Therefore, both Lj- *MxA* and Lj- *MxB* in heart and gill tissues participate in the immune response stimulated by Poly I:C and LPS, especially induced by Poly I:C. Because the response of Lj- *MxA* was more distinct than that of Lj- *MxB*, it is speculated that the two Mx copies may be involved in functional differentiation.

In summary, we cloned the homologous *L*. *japonica* proteins MxA and MxB, characterized their respective gene sequences, and assessed changes in their expression following challenge with Poly I:C and LPS. Structural and phylogenetic analyses revealed that the Lj-MxA and Lj-MxB protein sequences are highly conserved and may play a role in innate host defense. Changes in the Lj- *MxA* and Lj- *MxB* mRNA expression levels upon stimulation with LPS and Poly I:C indicated that these genes are important for antiviral immunity. However, differences in the structures of the Lj-MxA and Lj-MxB L4 loops and in the degree of immune response to stimuli suggested that functional differentiation occurred between the two Mx copies, possibly driven by positive selection. A total of 9 codons were found to be positively selected in Lj- *MxA* and Lj- *MxB*, most of which are concentrated in CID domains, suggesting that this structure may have an important function in adaptive evolution. Taken together, our findings show that Lj-MxA and Lj-MxB play crucial roles in defending fish against viral invasion. The exact details of the participation of Lj-MxA and Lj-MxB in immune defense require further study.

## Supporting information

378Supplementary_Materials

## Supplementary Data

Supplementary data is available at *Acta Biochimica et Biophysica Sinica* online.
