# Supplementary material for: Molecular evolution and expression patterns of myxovirus resistance proteins in Lampetra japonica : Evolution and expression of lamprey myxovirus resistance protein
Source: Acta Biochim Biophys Sin (Shanghai). 2024 Feb 23;56(3):490–3. doi: 10.3724/abbs.2024019 (PMC10984849; doi:10.3724/abbs.2024019)
Supplement: 378Supplementary_Materials [file 378Supplementary_Materials.pdf]

Supplementary Materials

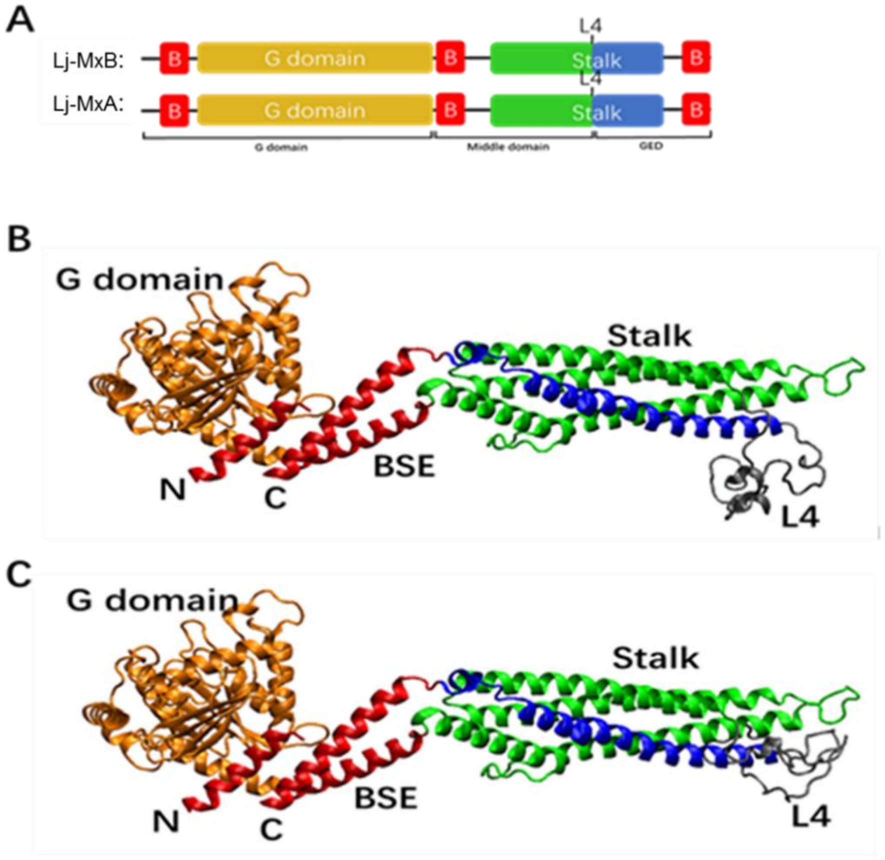

Supplementary Figure S1. Tertiary structure prediction of *L. japonica* MxA and MxB (A) Schematic diagrams of Lj-MxA and Lj-MxB structure. (B) Predicted tertiary structure of Lj-MxA. (C) Predicted tertiary structure of Lj-MxB.

Supplementary Table S1. Primers used in this study

Primer	Primer sequences (5′→3′)
Q-Lj-MxA-F	CCTCCAATTCCCACAAGTATCCG
Q-Lj-MxA-R	GCGATCTTGAAATATGAGCGCAG
Q-Lj-MxB-F	CAATCGGCAAAGGAAGAACCTC
Q-Lj-MxB-R	TCTCCTGGCTCTGAACCATCCT
Q-GAPDH-F	AACCAACTGCCTGGCTCCT
Q-GAPDH-R	GTCTTCTGCGTTGCCGTGT
Lj-MxA-F	CCGACGGCTTCATAACGAACTGT
Lj-MxA-R	TATGGAAGGAGCACAGGCAACAG
Lj-MxB-F	TGATGGAGCTGCGGTCTTCAT
Lj-MxB-R	TATGGAAGGAGCACAGGCAACAG

Supplementary Table S2. Accession number of Mxs for the construction of phylogenetic tree

Species	Gene name abbreviation	Accession number
Homo sapiens	Hs-MxA	NP_002453.2
Homo sapiens	Hs-MxB	NP_002454.1
Bos taurus	Bt-Mx1	NP_776365.1
Bos taurus	Bt-Mx2	NP_776366.1
Mus musculus	Mm-Mx1	NP_034976.1
Mus musculus	Mm-Mx2	NP_038634.1
Rattus norvegicus	Rn-Mx1	NP_775119.2
Rattus norvegicus	Rn-Mx2	NP_599177.2
Gallus gallus	Gg-Mx	NP_989940.2
Anser cygnoides	Ac-Mx	AND74630.1
Podarcis muralis	Po-Mx	XP_028583074.1
Xenopus tropicalis	Xt-Mx	XP_031752404.1
Lepisosteus oculatus	Lo-Mx1	XP_015219404.1
Lepisosteus oculatus	Lo-Mx2	XP_015219401.1
Lepisosteus oculatus	Lo-Mx3	XP_015196910.1
Danio rerio	Dr-MxA	NP_891987.2
Danio rerio	Dr-MxC	NP_001007285.1

Danio rerio	Dr-MxD	XP_695562.6
Danio rerio	Dr-MxF	XP_017206546.2
Eptatretus burgeri	Eb-Mx1	ENSEBUG00000002909
Eptatretus burgeri	Eb-Mx2	ENSEBUG00000002083.1
Eptatretus burgeri	Eb-Mx3	ENSEBUG00000001173.1
Callorhinchus milii	Cm-Mx1	XP_007904881.2
Lampetra morii	Lm-Mx	000218F: 434436-456308 bp[#]
Petromyzon marinus	Pm-Mx	XP_032804093.1
Branchiostoma floridae	Bf-Mx	XP_035690836.1
Saccoglossus kowalevskii	Sk-Mx	XP_006815062.1

[#]The sequence of *Lampetra morii* Mx was retrieved from an unpublished genome assembly with the gene location from 434436 bp to 456308 bp in scaffold 000218F.

Supplementary Table S3. Site-model and branch-site model to detect positive selection of Mx gene family

**Model**	**Parameter estimates**	**Model comparison**	**2ΔlnL(*P*-value)**	**Positive selection sites**[a]
**Site-model**				
M0:neutral	ω=0.22425			
M3:discrete	ω0=0.02851, *p*0=0.32394, ω1=0.22201, *p*1=0.40314, ω2=0.69294, *p*2=0.27292	M3 *vs* M0	3143.578376 (P < 0.001)	
M7:β	*p*=0.53418, *q*=1.28723			
M8:β and ω(β+ωS > 1)	*p*0=0.99999, *p*1=0.00001, ω=2.82171, *p*=0.26174, *q*=1.23926	M8 *vs* M7	801.713044 (P < 0.001)	Lj-MxA: 376S, 398K, 416R; Lj-MxB: 454S, 476K, 494R
**Branch-site model**				
MA0	ω0=0.22008 ω1=1.00000			
MA	p_0= 0.55504, p_1= 0.36570, p_{2a}= 0.04778, p_{2b}= 0.03148, Background ω_0=0.12987, ω_1=1, ω_{2a}= 0.12423, ω_{2b}= 1, Foreground ω_0=0.12987, ω_1=1, ω_{2a}= 6.89553, ω_{2b}= 6.89553	MA0 *vs* MA	2326.231372 (P < 0.001)	Lj-MxA:139K, 252R, 423W, 456A, 499A, 651A; Lj-MxB: 217K, 330R, 501S, 534V, 577A, 730L

[a]Only positively selected sites with Bayesian posterior probabilities over 95% are indicated.
